# I’ll Do It – After One More Scroll: The Effects of Boredom Proneness, Self-Control, and Impulsivity on Online Procrastination

**DOI:** 10.3389/fpsyg.2022.918306

**Published:** 2022-07-06

**Authors:** Cansu Sümer, Oliver B. Büttner

**Affiliations:** Economic and Consumer Psychology, Department of Computer Science and Applied Cognitive Science, University of Duisburg-Essen, Duisburg, Germany

**Keywords:** online procrastination, social media, boredom proneness, self-control, impulsivity, online shopping

## Abstract

Procrastination is a common phenomenon. With the increasing ubiquity of new media, research has started to investigate the ways in which these technologies are used as alternatives to task engagement. This paper extends the literature by examining procrastinatory uses of social media, instant messaging, and online shopping with respect to boredom proneness, self-control, and impulsivity among German and Turkish samples. Regression analyses revealed that boredom proneness, self-control, and the perseverance facet of impulsivity are especially significant predictors of online procrastination in both samples. The results between the two studies differ in terms of impulsivity. The findings of this paper highlight the thus far understudied role of boredom proneness and various aspects of impulsivity in online procrastination, and demonstrate that social media procrastination, instant messaging procrastination, and shopping procrastination tendencies likely have distinct underlying mechanisms.

## Introduction

Imagine the following scenario: You sit down to finally write that paper. You prepare everything you are going to need. You create a new document on your computer. You are all set but you don’t know where to start. You stare at the blank screen. Minutes go by. You tell yourself that you will find something to write while you are cleaning your desk, so you start organizing your workstation and think about the topic. Suddenly you get a new email from an old colleague. You wonder what they have been up to, so you check their Twitter profile. One thing leads to another, and you realize 3 hours have passed and you still haven’t written a single word.

Procrastination using the Internet has gained considerable attention recently. Online procrastination is associated with lower academic performance, higher negative affect, and negative self-evaluation (Lavoie and Pychyl, 2001; Reinecke and Hofmann, 2016; Troll et al., 2021). The Internet provides an instant access to pleasurable short-term activities and enables task postponement and immediate stress relief (Lavoie and Pychyl, 2001). To date, studies have focused on procrastination using Facebook (Meier et al., 2016) and general media (television, the Internet, smartphones; Lavoie and Pychyl, 2001; Schnauber-Stockmann et al., 2018). Indeed, Facebook and instant messaging are used for postponing studying, getting away from responsibilities, and putting tasks off (Quan-Haase and Young, 2010). Compared to their older counterparts, younger individuals use social networking sites (SNS) for procrastination more (Orchard et al., 2014).

Overall, literature indicates that online platforms are actively used as tools of procrastination. However, research is scarce regarding the procrastinatory uses of other common activities such as texting and online shopping. This is intriguing, given the reports of instant messaging applications such as WhatsApp being used regularly, delivering approximately 100 billion messages daily (Singh, 2020). Interestingly, some studies suggest that unconscientious individuals, who are more likely to procrastinate, tend to spend more time using WhatsApp (Montag et al., 2015). Although instant messaging is used frequently, no study to date has investigated whether it is indeed used for procrastinating. In a similar vein, online shopping has recently caught on, increasing by 19% in the last decade (Eurostat, 2022). Like social media, online shopping also provides an easy escape from work and everyday chores (Martínez-López et al., 2016), making it an attractive activity for procrastination. It is yet to be explored whether and how online shopping is used for procrastination as well. Therefore, the main goal of this paper was to seek an answer to how social media, instant messaging, and online shopping are used as tools of procrastination.

Both the general procrastination tendency and Internet use are influenced by proneness to get bored (Vodanovich and Rupp, 1999; Biolcati et al., 2018). However, it is unclear whether different forms of online procrastination are connected to boredom proneness. Thus, the second aim of this study was to investigate the effects of boredom proneness in addition to self-control and impulsivity as possible predictors of online procrastination. The contribution of this research is threefold. This paper is the first to distinguish between and examine different types of online procrastination (i.e., social media procrastination, instant messaging procrastination, and online shopping procrastination). Second, it is also the first to focus on the ways in which trait predictors (i.e., boredom proneness, self-control, impulsivity) contribute to these online procrastination tendencies. Finally, it strengthens its findings by examining these predictors across two culturally different samples (i.e., Germany and Turkey).

## Conceptual Background

Procrastination is the unnecessary postponing of the initiation or completion of an intended task despite the fact that the short-term prioritization of the delay will not outweigh the benefits of the long-term goals (Klingsieck, 2013). While students procrastinate more frequently than nonstudents (Svartdal et al., 2016), age is a more significant predictor of procrastination rather than one’s student status (Wypych et al., 2018). Indeed, age correlates negatively with general procrastination (Beutel et al., 2016) and academic procrastination (Beswick et al., 1988).

Literature approaches procrastination as a state variable (i.e., procrastination behavior over a specific period) or as a trait variable (i.e., general tendency to procrastinate). In this paper, we focus on procrastination as a trait in various domains (i.e., general tendency to procrastinate using social media, instant messaging, and online shopping, respectively). We examine three predictors of procrastination: boredom proneness, impulsivity, and self-control. The tendency to feel boredom regardless of the situation causes one to perceive even the most common tasks as requiring effort and makes one more likely to procrastinate in general (Farmer and Sundberg, 1986; Vodanovich and Rupp, 1999; Mercer-Lynn et al., 2014). Moreover, procrastination is often conceptualized as the result of a self-control conflict between short-term desires and long-term goals. Dual-process accounts conceptualize self-control conflicts as a battle between the impulsive and the reflective system: Whether individuals give in to the temptation to procrastinate or not depends on the predominance of either the reflective capacities for self-control or the automatic, impulsive tendencies (Hofmann et al., 2009, 2017). Hence, procrastination may be the result of high impulsivity, low self-control, or both. In the following, we review the three predictors boredom proneness, impulsivity, and self-control with regards to the general procrastination tendency and different forms of media use.

### Boredom

Boredom proneness is one construct that has been studied in relation to procrastination. Boredom is an aversive state where the individual is unable to engage their attention to the stimulus, is aware of this inability, and they ascribe the environment as the cause (Eastwood et al., 2012). Both attentional failures and a lack of perceived meaningfulness can lead to feelings of boredom (Westgate and Wilson, 2018). Irrespective of the situation, individuals with higher boredom proneness experience boredom more frequently, more intensely, and perceive their lives as more boring (Mercer-Lynn et al., 2014; Tam et al., 2021). They also perceive even the most typical tasks as requiring effort and tend to procrastinate more (Farmer and Sundberg, 1986; Blunt and Pychyl, 1998; Vodanovich and Rupp, 1999; Ferrari, 2000). Therefore, when people get bored during a task or have a stronger propensity to get bored in general, they are more likely to procrastinate.

It is argued that, as an adaptive state, boredom signals that one’s current situation is no longer stimulating enough and thus it urges pursuing alternative activities (Bench and Lench, 2013). When individuals are bored, they frequently turn to their smartphones and social media as a pastime and to procrastinate (Martin et al., 2006; Blight et al., 2017; Alblwi et al., 2019; Koessmeier and Büttner, 2021). Similarly, people with higher boredom proneness use smartphones, SNS, and instant messaging applications more frequently (Matic et al., 2015; Wegmann et al., 2018). Shopping is also viewed as an escape from everyday life (Parsons, 2002). In fact, boredom is a strong motivation for visiting online stores and shopping impulsively, as higher boredom proneness leads to more impulse purchases (Sundstrom et al., 2019; Bozaci, 2020). To cope with boredom, individuals visit online stores and place items on their online shopping carts without any intention of buying (Kukar-Kinney and Close, 2010).

Given that both procrastination and boredom involve the urge to alleviate unpleasant states and that online services, such as social media and online stores, are frequently used to relieve boredom, it is surprising that no study has investigated whether boredom proneness is related to online procrastination. Thus, we addressed this gap by examining the ways in which boredom proneness is related to procrastinatory social media use, instant messaging, and online shopping, and expected that

H1: Boredom proneness is positively related to all types of online procrastination.

### Impulsivity

Impulsivity is another predictor of trait procrastination. While an impulse is a strong, specific, and automatically triggered inclination to approach or act on an immediate temptation or toward their short-term gratifications (Hofmann et al., 2009), impulsivity is a multifaceted construct. Specifically, Whiteside and Lynam’s (2001) framework highlights four separate aspects of impulsivity. The first of these is perseverance, which describes the capacity to begin and stay focused on a task until its completion. The premeditation facet concerns the ability to consider the consequences of one’s actions beforehand. Sensation seeking refers to openness to pursue new activities. Finally, the urgency facet is the tendency to act rashly when experiencing negative emotions. Apart from sensation seeking, all impulsivity factors seem to be related to procrastination. That is, while urgency positively relates to the general procrastination tendency, premeditation and perseverance are negatively related to it (Rebetez et al., 2018). Overall, a lack of perseverance or a lower capacity to remain focused on a task until its completion is the strongest predictor of procrastination (Wypych et al., 2018). In addition to this multifaceted framework of impulsivity, alternative or composite conceptualizations of this construct, such as a trait that “indicates spontaneity and a tendency to act upon whims and inclinations,” are also associated with the general procrastination tendency (Steel, 2007, p. 69).

In general, higher impulsivity is associated with problematic behaviors, such as problematic uses of the smartphone, instant messaging apps, and social media (Billieux et al., 2008b; Sindermann et al., 2020). Individuals with higher impulsivity use instant messaging more automatically and for longer hours (Levine et al., 2013; Bayer et al., 2016) and more specifically, those with higher urgency send more SMSs daily (Billieux et al., 2008b). Finally, as an aspect of impulsivity, higher urgency is also the only predictor of compulsive buying (Billieux et al., 2008a). In summary, higher impulsivity and more specifically a heightened urgency is one predictor of actual and problematic media use and shopping.

Research is limited on whether impulsivity is related to online procrastination. By following Whiteside and Lynam’s (2001) impulsivity framework, we aimed to examine the effects of different aspects of this trait on online procrastination. Literature suggests that lower perseverance is the strongest predictor of procrastination in general. We had no a priori hypotheses about which aspects of impulsivity would be more important for specific types of online procrastination. Therefore, we expected that

H2: The perseverance aspect of impulsivity is negatively related to all types of online procrastination.

### Self-Control

Self-control is the ability to willfully adjust behaviors when one’s abstract or remote goals (e.g., getting good grades) are conflicted by more concrete or immediate desires (e.g., going on Instagram to see what is new) and to refrain from acting on the latter (Tangney et al., 2004; Fujita, 2011). In conditions where the strength of the impulsive system increases, the reflective system may fail to inhibit and override impulses, whose “activation level exceeds the critical threshold necessary for the execution of self-controlled behavior” (Hofmann et al., 2009, p. 165). This capacity to successfully deal with problematic desires that conflict with one’s goals is crucial for task completion and procrastination (Sirois and Pychyl, 2013; Pychyl and Sirois, 2016). Indeed, self-control is negatively related to the general procrastination tendency (Wijaya and Tori, 2018). In short, self-control prevents one from giving in to the temptation of quitting the task in favor of more pleasant alternatives or not engaging with it at all.

The ever-present availability of media poses a challenge for media consumers’ goals and task completion in everyday life (Hofmann et al., 2017), which is why self-control is one of the constructs that have been most commonly examined in relation to media use. Specifically, self-control is negatively associated with habitual Facebook checking as well as the duration of media use, including daily instant messaging, SNS, TV, and online videos (Panek, 2014; Li et al., 2016; Meier et al., 2016). Lower self-control also predicts problematic online shopping and compulsive buying (Achtziger et al., 2015; Jiang et al., 2017). Thus, difficulties with successfully handling desires for media use in favor of higher-order goals can lead to procrastination.

Similar to the negative associations between self-control and general procrastination tendency (Wijaya and Tori, 2018), research indicates that self-control is negatively related to procrastinatory uses of Facebook (Meier et al., 2016), smartphones (Schnauber-Stockmann et al., 2018; Troll et al., 2021), and general media (the Internet, TV, video games; Reinecke and Hofmann, 2016). Similarly, ego depletion, that is, the “temporary reduction in the self’s capacity or willingness to engage in volitional action” (Baumeister et al., 1998, p. 1253), is positively associated with procrastinatory media use (Reinecke et al., 2014). Overall, these studies suggest that individuals with lower self-control are more likely to use media to procrastinate. Therefore, we also examined the effect of self-control on different types of online procrastination and expected that

H3: Self-control is negatively related to all types of online procrastination.

### Overview of Studies

To address these, two online studies were carried out. Study 1 used a quota sample and investigated how people use online services for procrastination in Germany. It also aimed to establish the individual differences in trait self-control, boredom proneness, and impulsivity in relation to different types of online procrastination. Because we wanted to explore the differences between the predictors of online procrastination across different countries, we carried out Study 2 using the same measures in a convenience sample from a different cultural background, namely, Turkey. We did not have a priori hypotheses regarding these differences and addressed this research question in an exploratory way.

## Study 1

## Materials and Methods

### Sample

Data was collected from 333 German participants through a commercial online access panel. As we aimed for a heterogeneous sample that reflects individuals with different backgrounds and experiences, we used quotas for gender and age (50% men, 50% women; 20% from each age group: 18–29, 30–39, 40–49, 50–59, 60–69 years). Additionally, we wanted to focus on individuals that were active users of social media and instant messaging. Thus, to be eligible, participants had to have at least one social media or instant messaging account and use social media at least a few times a month. Participants who finished the survey in less than 3 min, failed to complete it in a single session, and failed both control questions were excluded. The final dataset included 307 participants (*M*_age_ = 44.66 years, SD = 14.58, 49.8% female; see Table 1 for participant demographics).

**TABLE 1:** Participant demographics.

		%	
		German	Turkish
Age	18–29	18.6	22.9
	30–39	19.9	27.3
	40–49	20.5	21.0
	50–59	19.9	20.0
	60–69	21.1	8.8
Gender	Male	50.2	30.2
	Female	49.8	67.8
	Other		2.0
Employment status	Employee	51.5	43.9
	In training	10.7	16.1
	Self-employed or part-time	5.2	9.3
	Job seeking	4.6	14.6
	Active in the household, retired or other	28.0	16.1
Marital status	Single	28.0	41.5
	Married or in a partnership	58.6	46.3
	Divorced, widowed or other	13.4	12.2

### Measures

In addition to demographics information, participants reported how many hours they spent on social media and on the Internet, daily. Finally, they reported on the following scales.

#### Online Procrastination

To our knowledge, there are no standardized scales of online procrastination. Therefore, the four-item measure used by Reinecke et al. (2014) was adapted to measure procrastinatory social media use (e.g., “I use social media although I have planned to get something done”), instant messaging (e.g., “I use instant messaging although I have more important things to do”), and browsing of online shops (e.g., “I browse online shops while procrastinating upcoming work”), separately. The German translations were adapted from Troll et al. (2021). The items were rated on a five-point rating scale (αs = 0.95 for all three types of online procrastination).

#### Boredom Proneness

The eight-item Short Boredom Proneness Scale (SBPS; Farmer and Sundberg, 1986; Struk et al., 2017) was used to measure the tendency to experience boredom (e.g., “I find it hard to entertain myself”). The SBPS was translated into German by Martarelli et al. (2020). The items were rated on a seven-point rating scale (α = 0.91).

#### Trait Self-Control

The Brief Self-Control Scale (Tangney et al., 2004) is a widely used measure of trait self-control (e.g., “I’m good at resisting temptation”). It was adapted to German by Bertrams and Dickhäuser (2009) and included 13 items, which were rated on a five-point rating scale (α = 0.84).

#### Impulsivity

The UPPS Impulsive Behavior Scale was created by Whiteside and Lynam (2001) to capture the four facets of impulsivity through the subscales Urgency, Premeditation, Perseverance, and Sensation Seeking. A 20-item short version that includes the four subscales was adapted to German by Keye et al. (2009). The items were rated on a five-point scale (α = 0.80 for Urgency, 0.69 for Premeditation, 0.72 for Perseverance, 0.75 for Sensation Seeking).

#### Trait Procrastination

The Pure Procrastination Scale was used to measure chronic procrastination (e.g., “I’m continually saying ‘I’ll do it tomorrow”’; Steel, 2010). It was adapted to German by Svartdal et al. (2016) and included 12 items, which were rated on a five-point rating scale (α = 0.91).

## Results

Means and standard deviations can be found in Table 2. In order to determine whether or not to include age and gender as control variables in our further analyses, we carried out independent samples *t*-test and discovered gender differences in sensation seeking, with men (*M* = 2.60, SD = 0.92) scoring higher than women (*M* = 2.31, SD = 0.93), *t*(305) = −2.67, *p* < 0.01. Younger individuals used social media longer (*r* = −0.248, *p* < 0.001). The correlation between age and hours spent online was marginally significant (*r* = −0.104, *p* = 0.069). For exploratory purposes, we examined the correlations between trait procrastination and other variables. Trait procrastination had a higher correlation with social media procrastination (*r* = 0.68, *p* < 0.01) than with instant messaging procrastination (*r* = 0.59, *p* < 0.01) and shopping procrastination (*r* = 0.58, *p* < 0.01). See Table 3 for further bivariate correlations. Further exploratory analyses showed that Facebook, Instagram, and YouTube were the most frequently visited websites that were used while procrastinating (see Table 4).

**TABLE 2:** Means and standard deviations of variables.

	German	Turkish
	
	*M* (SD)	*M* (SD)
Age	44.66 (14.58)	40.13 (13.18)
Online hours	4.17 (2.82)	4.91 (3.09)
Social media hours	2.10 (2.27)	2.81 (2.34)
Self-control	3.36 (0.63)	3.31(0.69)
UPPS: Urgency	2.33 (0.76)	2.63 (0.75)
UPPS: Premeditation	3.68 (0.61)	3.94 (0.61)
UPPS: Perseverance	3.86 (0.64)	3.53 (0.77)
UPPS: Sensation Seeking	2.45 (0.93)	2.86 (0.96)
Trait procrastination	2.16 (0.73)	2.77 (0.90)
Boredom proneness	2.54 (1.16)	3.30 (1.47)
Social media procrastination	2.28 (0.97)	2.80 (1.09)
Instant messaging procrastination	2.16 (0.93)	2.83 (1.07)
Shopping procrastination	2.04 (0.90)	1.93 (0.90)

**TABLE 3:** Bivariate correlations in the German sample above the diagonal and the Turkish below the diagonal.

	1	2	3	4	5	6	7	8	9	10	11	12	13
1. Age		–0.10	−0.24**	0.24**	−0.21**	0.05	0.23**	−0.27**	−0.28**	−0.28**	−0.43**	−0.43**	−0.28**
2. Online hours	−0.36**		0.38**	–0.10	0.04	–0.01	–0.09	0.05	–0.00	0.11*	0.09	0.06	0.02
3. Social media hours	−0.22**	0.64**		−0.21**	0.24**	0.01	−0.18**	0.13*	0.14*	0.22**	0.31**	0.24**	0.24**
4. Self-control	0.50**	−0.40**	−0.27**		−0.61**	0.30**	0.67**	−0.15**	−0.62**	−0.62**	−0.53**	−0.51**	−0.45**
5. UPPS: Urgency	−0.34**	0.37**	0.25**	−0.66**		−0.27**	−0.56**	0.23**	0.45**	0.55**	0.42**	0.44**	0.43**
6. UPPS: Premeditation	–0.05	–0.03	–0.05	0.31**	−0.32**		0.36**	–0.05	−0.15**	−0.11*	–0.08	–0.08	–0.10
7. UPPS: Perseverance	0.46**	−0.32**	−0.21**	0.73**	−0.48**	0.29**		−0.14*	−0.68**	−0.65**	−0.51**	−0.45**	−0.48**
8. UPPS: Sensation Seeking	−0.28**	0.13*	0.04	−0.25**	0.21**	0.02	–0.08		0.15**	0.16**	0.23**	0.18**	0.17**
9. Trait procrastination	−0.41**	0.40**	0.27**	−0.70**	0.48**	–0.11	−0.73**	0.09		0.65**	0.68**	0.59**	0.58**
10. Boredom proneness	−0.48**	0.38**	0.25**	−0.58**	0.49**	–0.09	−0.59**	0.15*	0.65**		0.59**	0.51**	0.48**
11. Social media procrastination	−0.46**	0.51**	0.36**	−0.61**	0.44**	–0.11	−0.61**	0.12	0.71**	0.54**		0.78**	0.63**
12. Instant messaging procrastination	−0.37**	0.35**	0.22**	−0.53**	0.38**	–0.09	−0.50**	0.15*	0.54**	0.48**	0.68**		0.65**
13. Shopping procrastination	−0.28**	0.32**	0.14*	−0.42**	0.37**	–0.06	−0.34**	0.01	0.41**	0.41**	0.44**	0.37**	

*UPPS, UPPS Impulsive Behavior Scale.*

**p < 0.01.

*p < 0.05.

**TABLE 4:** Percentage of social media sites and instant messaging applications that are used for procrastination.

		German	Turkish
Social media sites	Facebook	51.8	42.0
	Instagram	33.2	66.3
	Twitter	9.4	38.5
	YouTube	45.0	57.1
	Tumblr	2.0	18.5
	Snapchat	7.8	2.0
	Pinterest	1.0	13.7
	Other	14.0	18.5
	None	19.9	2.0
Instant messaging	WhatsApp	77.9	92.7
	Facebook Messenger	21.5	17.1
	Telegram	8.8	13.2
	Instagram Messenger	0.3	1.5
	Other	11.8	6.8
	None	18.9	4.4

To test the hypotheses, three hierarchical regression analyses were carried out with procrastinatory social media use, instant messaging, and visits to online shops as the dependent variable, separately. In all three analyses, boredom proneness, self-control, and the four impulsivity facets were entered as predictors in Step 1. Age and gender were entered as control variables in Step 2.

For social media procrastination, the final model showed that boredom proneness was the strongest predictor (β = 0.30, *p* < 0.001), followed by age (β = −0.24, *p* < 0.001), self-control (β = −0.20, *p* = 0.001), and perseverance (β = −0.13, *p* = 0.044). For procrastinatory instant messaging, age was the strongest predictor (β = −0.27, *p* < 0.001), followed by self-control (β = −0.22, *p* = 0.001), boredom proneness (β = 0.17, *p* = 0.008), and urgency (β = 0.12, *p* = 0.033). Finally, for procrastinatory browsing of online stores, perseverance (β = −0.22, *p* = 0.003) made the strongest contribution, followed by boredom proneness (β = 0.16, *p* = 0.019), urgency (β = 0.12, *p* = 0.049), and age (β = −0.11, *p* = 0.025). The detailed results of the regression analyses are available in Table 5.

**TABLE 5:** Hierarchical regressions predicting online procrastination in the German sample.

	Procrastinatory social media				Procrastinatory instant messaging				Procrastinatory shopping			
Variable	*B*	SE	β	*p*	*B*	SE	β	*p*	*B*	SE	β	*p*
**Step 1**												
Constant	2.74	0.58		<0.001	2.37	0.60		<0.001	2.54	0.59		<0.001
Boredom proneness	0.29	0.05	0.34	<0.001	0.18	0.05	0.22	0.001	0.14	0.05	0.18	0.008
Self-control	–0.36	0.10	–0.23	<0.001	–0.37	0.10	–0.25	<0.001	–0.18	0.10	–0.12	0.077
UPPS: Urgency	0.00	0.07	0.00	0.98	0.15	0.07	0.12	0.046	0.15	0.07	0.12	0.050
UPPS: Sensation Seeking	0.12	0.04	0.12	0.006	0.07	0.04	0.07	0.114	0.06	0.04	0.07	0.148
UPPS: Perseverance	–0.20	0.10	–0.13	0.046	–0.11	0.10	–0.08	0.258	–0.31	0.10	–0.22	0.003
UPPS: Premeditation	0.13	0.07	0.08	0.074	0.13	0.07	0.09	0.082	0.11	0.07	0.08	0.130
*R* ^2^	0.428				0.342				0.318			
Adjusted *R*^2^	0.416				0.333				0.304			
**Step 2**												
Constant	3.69	0.58		<0.001	3.40	0.59		<0.001	3.01	0.61		<0.001
Boredom proneness	0.25	0.05	0.30	<0.001	0.14	0.05	0.17	0.008	0.12	0.05	0.16	0.019
Self-control	–0.32	0.09	–0.20	0.001	–0.32	0.10	–0.22	0.001	–0.15	0.10	–0.11	0.123
UPPS: Urgency	0.00	0.07	0.00	0.970	0.15	0.07	0.12	0.033	0.15	0.07	0.12	0.049
UPPS: Sensation Seeking	0.08	0.04	0.07	0.088	0.02	0.04	0.02	0.617	0.05	0.04	0.05	0.299
UPPS: Perseverance	–0.19	0.09	–0.13	0.044	–0.11	0.10	–0.07	0.261	–0.31	0.10	–0.22	0.003
UPPS: Premeditation	0.13	0.07	0.08	0.069	0.13	0.07	0.08	0.075	0.11	0.07	0.08	0.128
Age	–0.01	0.00	–0.24	<0.001	–0.01	0.00	–0.27	<0.001	–0.00	0.00	–0.11	0.025
Gender	–0.11	0.08	–0.05	0.186	–0.12	0.08	–0.06	0.145	–0.09	0.08	–0.05	0.297
*R* ^2^	0.484				0.41				0.333			
Adjusted *R*^2^	0.470				0.40				0.315			

In summary, boredom proneness was positively related to social media procrastination, instant messaging procrastination, and online shopping procrastination. Thus, H1 is supported. Perseverance was negatively related to social media procrastination and online shopping procrastination, but not instant messaging procrastination. Hence, H2 is partially supported. Finally, self-control was negatively related to social media procrastination, instant messaging procrastination, but not shopping procrastination. Therefore, H3 is also only partially supported.

## Discussion

Study 1 examined the effects of several personality traits on online procrastination in a German sample. The positive correlations between general procrastination tendency and different types of online procrastination suggest that chronic procrastinators also use social media, instant messaging, and online shopping for procrastination. Regression analyses showed that younger and more boredom prone individuals used social media, instant messaging, and online shopping more frequently to procrastinate. Moreover, individuals with lower self-control used social media and instant messaging more to procrastinate. Finally, urgency predicted procrastinatory instant messaging and online shopping, whereas perseverance predicted procrastinatory social media use and online shopping. In Study 2, we investigated the effects of these predictors in another country.

## Study 2

## Materials and Methods

### Sample

In total, 217 Turkish adults participated. Participants were reached through snowballing and word-of-mouth. Again, participants had to have at least one social media or instant messaging account and use social media at least a few times a month. The final dataset included 205 participants (*M*_age_ = 40.13, SD = 13.18, 67.8% female).

### Measures

We used the same measures as in Study 1 in Turkish versions. For online procrastination, the four-item measure from Reinecke et al. (2014) was used again to measure procrastination with social media, instant messaging, and online shopping, separately. The items were translated by the first author and reviewed by an English-Turkish translator (αs between 0.94 and 0.96). For boredom proneness, we used the Turkish version of the short SBPS (Güner et al., 2021; α = 0.90). For trait self-control, BSCS was used in Turkish (Nebioglu et al., 2012; α = 0.85). The UPPS Impulsive Behavior Scale was used in Turkish (Yargıç et al., 2011; α = 0.71 for Urgency, 0.71 for Premeditation, 0.77 for Perseverance, 0.78 for Sensation Seeking). For trait procrastination, we used the Turkish version of the PPS (Balkis and Duru, 2019; α = 0.92).

## Results

Independent samples *t*-tests indicated significant gender differences in sensation seeking, with men (*M* = 3.47, SD = .78) scoring higher than women (*M* = 2.59, SD = 0.93), *t*(138) = −6.96, *p* < 0.001. On self-control, women (*M* = 3.41, SD = 0.68) scored higher than men (*M* = 3.12, SD = 0.68), *t*(199) = 2.74, *p* < 0.01. Younger individuals spent longer using both social media (*r* = −0.224, *p* = 0.000) and the Internet (*r* = −0.369, *p* < 0.000). Trait procrastination had a higher correlation with social media procrastination (*r* = 0.71, *p* < 0.01) than with instant messaging (*r* = 0.54, *p* < 0.01) and shopping (*r* = 0.41, *p* < 0.01). See Table 3 for further bivariate correlations.

The same hierarchical analyses were carried out as in Study 1. Specifically, three hierarchical regression analyses were carried out with procrastinatory social media use, instant messaging, and visits to online shops as the dependent variable, separately. In all analyses, boredom proneness, self-control, and the four impulsivity facets were entered as predictors in Step 1. Age and gender were entered as control variables in Step 2.

For social media procrastination, in the final model, perseverance was the strongest predictor (β = −0.28, *p* = 0.001), followed by self-control (β = −0.24, *p* = 0.012), and boredom proneness (β = 0.15, *p* = 0.029). For procrastinatory instant messaging, self-control (β = −0.27, *p* = 0.009) and boredom proneness (β = 0.18, *p* = 0.019) were the only significant predictors. For procrastinatory shopping, self-control (β = −0.28, *p* = 0.011) made the strongest contribution, followed by boredom proneness (β = 0.18, *p* = 0.030), and gender (β = −0.15, *p* = 0.026). The detailed results of the regression analyses are available in Table 6.

**TABLE 6:** Hierarchical regressions predicting online procrastination in the Turkish sample.

	Procrastinatory social media				Procrastinatory instant messaging				Procrastinatory shopping			
Variable	*B*	SE	β	*p*	*B*	SE	β	*p*	*B*	SE	β	*p*
**Step 1**												
Constant	4.51	0.746		<0.001	4.08	0.818		<0.001	1.94	0.739		0.009
Boredom proneness	0.13	0.05	0.17	0.011	0.14	0.05	0.19	0.013	0.13	0.05	0.21	0.008
Self-control	–0.43	0.14	–0.27	0.003	–0.45	0.16	–0.29	0.005	–0.36	0.14	–0.27	0.014
UPPS: Urgency	0.08	0.10	0.05	0.428	0.02	0.11	0.02	0.803	0.17	0.10	0.14	0.083
UPPS: Sensation Seeking	–0.01	0.06	–0.01	0.769	0.03	0.06	0.03	0.575	–0.11	0.06	–0.12	0.065
UPPS: Perseverance	–0.44	0.11	–0.31	<0.001	–0.26	0.12	–0.18	0.042	0.03	0.11	0.02	0.781
UPPS: Premeditation	0.17	0.10	0.10	0.080	0.13	0.11	0.07	0.220	0.12	0.10	0.08	0.224
*R* ^2^	0.471				0.350				0.248			
Adjusted *R*^2^	0.455				0.330				0.225			
**Step 2**												
Constant	4.91	0.798		<0.001	4.18	0.882		<0.001	2.45	0.78		0.002
Boredom proneness	0.11	0.05	0.15	0.029	0.13	0.05	0.18	0.019	0.11	0.05	0.18	0.030
Self-control	–0.37	0.14	–0.24	0.012	–0.43	0.16	–0.27	0.009	–0.37	0.14	–0.28	0.011
UPPS: Urgency	0.07	0.10	0.05	0.441	0.02	0.11	0.02	0.807	0.17	0.10	0.14	0.092
UPPS: Sensation Seeking	–0.04	0.06	–0.04	0.461	0.02	0.07	0.02	0.755	–0.07	0.06	–0.07	0.289
UPPS: Perseverance	–0.40	0.11	–0.28	0.001	–0.24	0.13	–0.17	0.060	0.02	0.11	0.01	0.865
UPPS: Premeditation	0.12	0.10	0.07	0.223	0.11	0.11	0.06	0.310	0.13	0.10	0.08	0.201
Age	–0.01	0.00	–0.12	0.054	–0.00	0.00	–0.04	0.560	–0.00	0.00	–0.04	0.547
Gender	0.03	0.12	0.01	0.755	0.03	0.13	0.01	0.782	–0.26	0.11	–0.15	0.026
*R* ^2^	0.481				0.351				0.268			
Adjusted *R*^2^	0.460				0.325				0.238			

In summary, boredom proneness was positively related to all three types of online procrastination. Thus, H1 is supported. Perseverance was negatively related to social media procrastination but not instant messaging procrastination or shopping procrastination. Hence, H2 is only partially supported. Finally, self-control was negatively related to all types of online procrastination. Therefore, H3 is supported.

## Discussion

Study 2 aimed to explore the effects of the same predictors in Study 1 in a different sample. As earlier, higher boredom proneness and lower self-control and perseverance predicted social media procrastination. For instant messaging procrastination, higher boredom and lower self-control increased this tendency. Unlike Study 1, in which urgency was a positive predictor, no impulsivity facet predicted this procrastination tendency. Finally, in Study 1, boredom proneness, urgency, and perseverance had predicted shopping procrastination. In Study 2, boredom proneness and self-control were the only significant predictors of this tendency.

## General Discussion

The aim of this paper was to extend the online procrastination literature by investigating the effects of self-control, boredom proneness, and impulsivity. We focused on procrastinatory social media use, instant messaging, and browsing of online stores in German and Turkish samples. Our findings demonstrate that higher boredom proneness, lower self-control, and lower perseverance are especially predictive of different types of online procrastination tendencies across both samples. To our knowledge, this study is the first attempt to understand the role of boredom proneness in online procrastination. We found that a higher propensity to get bored leads to more frequent procrastination with social media, instant messaging apps, and online shops. While some trait variables (i.e., boredom proneness and self-control) predicted all three types of online procrastination, others (e.g., premeditation) did not influence any of these tendencies, indicating that these trait variables have separate predictive values for different types of online procrastination, and that social media procrastination, instant messaging procrastination, and shopping procrastination have distinct underlying processes and should be considered separately. Accordingly, we will first focus on boredom proneness and self-control, as all types of online procrastination were predicted by these constructs. Then, we will discuss the differences between the types of online procrastination regarding separate aspects of impulsivity. Finally, we will turn to the differences between our samples.

### Boredom Proneness and Self-Control

As expected, boredom proneness was positively related to all types of online procrastination in both studies. Specifically, individuals with stronger propensity to get bored tended to use social media, instant messaging, and online shopping for procrastination more. This is in line with previous research that shows that these platforms provide an attractive alternative to procrastinate with when individuals get bored (Alblwi et al., 2019). Indeed, social media and online shopping can provide a relief from boredom (Kukar-Kinney and Close, 2010; Zolkepli and Kamarulzaman, 2015). Frequent social media use, instant messaging, and online shopping has been shown to be associated with higher boredom proneness (Matic et al., 2015; Wegmann et al., 2018). Accordingly, we found that having a higher tendency to get bored regardless of one’s situation increases the likelihood to use social media and instant messaging and browse online stores rather than engage in the current task. These findings indicate that boredom proneness contributes to different types of online procrastination in addition to self-control and impulsivity.

Each type of online procrastination was also predicted by self-control. While the negative association between self-control and social media procrastination replicates prior research (Meier et al., 2016), our findings regarding procrastinatory instant messaging and shopping are novel. Specifically, individuals with lower self-control used social media, instant messaging, and online shopping for procrastination more frequently. These results support prior studies. The wish to use media is one of the most frequently experienced desires and is also amongst the desires that are most frequently surrendered to (Hofmann et al., 2012). Indeed, self-control is negatively associated with problematic media use and online shopping (Panek, 2014; Jiang et al., 2017). Having a lower capacity for “overriding prepotent responses (e.g., impulses or habits)” and refraining from acting on them results in failures of self-control (Hofmann et al., 2009, p. 165). In line with these findings, our results indicate that having lower levels of self-control capabilities increases the tendency to use social media platforms, send instant messages, and visit online stores to postpone one’s tasks.

### Impulsivity

Our results regarding the relationships between impulsivity and different types of online procrastination were mixed. Although the correlations between the three types of online procrastination and all impulsivity facets (except premeditation) were significant, perseverance and urgency were the only facets that predicted different types of online procrastination. Perseverance, that is, the ability to stay focused on a task until its completion, was negatively related to social media procrastination in both studies. No other facet of impulsivity was significant for procrastinatory social media use. Perseverance is associated with the capacity to hold back irrelevant thoughts as well as with trait procrastination (Bechara and Van der Linden, 2005; Rebetez et al., 2018). Individuals with lower perseverance capacities may be tempted to use their smartphones due to these irrelevant thoughts (Billieux et al., 2008b). Similarly, we found that lower perseverance increases the likelihood to use social media as tools of procrastination.

In contrast, perseverance did not influence instant messaging procrastination. While we did not have a priori expectations about other impulsivity facets than perseverance, urgency predicted procrastinatory instant messaging in Study 1, which implies different underlying mechanisms between these procrastination tendencies. Specifically, higher tendency to act impulsively when experiencing unpleasant emotional states, namely urgency, increased the likelihood to use instant messaging for procrastination. This aspect of impulsivity is associated with the number of daily SMSs sent, suggesting that, for individuals that feel like they need to pursue their impulses at once, texting can be an ideal solution when they are feeling down (Billieux et al., 2008b). Instant messaging enables communication with close others in distressing times (Cui, 2016). Indeed, students postpone assignments by first texting their friends and sharing their negative feelings (Deng, 2020). Yet, the urge to check for new online messages can contribute to daily procrastination (Meier, 2021). Our results demonstrate that higher tendency to act rash when feeling negative emotions increases procrastination with instant messaging.

Finally, we investigated procrastinatory browsing of online stores and found that perseverance and urgency were associated with it in Study 1. Specifically, lower perseverance and higher urgency simultaneously increased the tendency to visit online stores for procrastination. This is partly in line with the literature. Window-shopping can uplift consumers’ moods, which is a strong motivation for online impulse purchases (Woodruffe, 1997; Sundstrom et al., 2019) and urgency is the only predictor of compulsive buying (Billieux et al., 2008a). The significance of perseverance in our results implies that, in addition to urgency, a lower capacity to remain focused on a task also increases procrastinatory shopping tendencies. Individuals who experience difficulties with staying concentrated on a task may also struggle with inhibiting task-irrelevant thoughts (e.g., a sale in a clothing store) and be more likely to browse online stores to procrastinate.

### Sample Differences

Literature indicates that cultural factors affect both media use and procrastination (Mann et al., 1998; Goodrich and de Mooij, 2014). Accordingly, our two studies differed in the effects of certain predictors. Age negatively influenced all types of online procrastination for the Germans, such that, younger Germans were more likely to use the Internet for procrastination. This is in line with Beutel et al. (2016), who found that trait procrastination decreased with age across German samples. Although these behaviors did decrease with age with our Turkish participants, it did not predict procrastination, which replicates past findings in Turkey (Bekleyen, 2017). Alternatively, the restricted age range and variance in the Turkish sample might have prevented age from becoming a significant predictor, although in both samples younger individuals used social media, instant messaging, and online shopping for procrastination more than their older counterparts.

We further found that, for the Germans but not the Turkish, perseverance predicted shopping procrastination and urgency predicted instant messaging and shopping procrastination. These cultural differences in the effect of impulsivity resemble prior research, which indicate that culture has an influence on actual and problematic (online) shopping behaviors and the frequency of visiting online stores (Gong, 2009; Baron and af Segerstad, 2010). Overall, for the Turkish, impulsivity does not make any significant contribution while predicting procrastinatory instant messaging and shopping as it does for the Germans.

### Limitations

One limitation of this study is its correlational design, which precludes definite causal inferences. Longitudinal studies should clarify the directionality between boredom proneness and media use. Moreover, we did not differentiate between devices (e.g., smartphone, computer) that were used when accessing these platforms, although social media sites are accessed increasingly more through smartphones and tablets compared to personal computers (Droesch, 2019). Future studies on online procrastination could delve into possible differences between the mobile devices and computers as tools of procrastination. Furthermore, as some studies indicate that social media can be used both to escape unpleasant life situations as well as to procrastinate (Meier et al., 2018), the role of escapism in online procrastination should be further explored. Finally, we need to consider the impact of the COVID-19 pandemic, during which most people worked from home. Our results might also be influenced by factors such as lower structure and motivation (Melgaard et al., 2022).

## Conclusion

Online procrastination is an increasingly common phenomenon. Literature has investigated the uses of general media, Facebook, and smartphones for procrastination. The purpose of this paper was to better understand the connections between several personality traits and types of online procrastination. Accordingly, we examined the influence of boredom proneness, self-control, and impulsivity on procrastinatory social media use, instant messaging, and online shopping tendencies. Our results show that, in addition to self-control, boredom proneness is especially predictive of online procrastination.

## Data Availability Statement

The datasets presented in this study can be found in online repositories. The names of the repository and accession number can be found at: https://osf.io/czbka/.

## Ethics Statement

The studies involving human participants were reviewed and approved by Ethics Committee of the Department of Computer Science and Applied Cognitive Science, University of Duisburg-Essen, Duisburg, Germany. The participants provided their written informed consent to participate in this study.

## Author Contributions

CS and OB designed the study. CS organized data collection, performed the statistical analysis, and wrote the first draft of the manuscript. CS and OB revised the manuscript and approved the submitted version. Both authors contributed to the article and approved the submitted version.

## Conflict of Interest

The authors declare that the research was conducted in the absence of any commercial or financial relationships that could be construed as a potential conflict of interest.

## Publisher’s Note

All claims expressed in this article are solely those of the authors and do not necessarily represent those of their affiliated organizations, or those of the publisher, the editors and the reviewers. Any product that may be evaluated in this article, or claim that may be made by its manufacturer, is not guaranteed or endorsed by the publisher.
